# An injectable thermosensitive PLGA–PEG–PLGA hydrogel integrated with coordination-driven self-assembled MTX–Mn nanoparticles for enhanced melanoma therapy *via* mitochondrial dysfunction

**DOI:** 10.1039/d6ra01096b

**Published:** 2026-04-22

**Authors:** Xiaolong Xu, Yuze Zhou, Junhao Chen, Lujing Fei, Min Qi

**Affiliations:** a Department of Plastic Surgery, Xiangya Hospital, Central South University Changsha 410028 China; b Department of Dermatology, Shenzhen People's Hospital, The Second Clinical Medical College, Jinan University Shenzhen 518020 China; c Department of Burns and Plastic Surgery, Shenzhen Hospital of Southern Medical University, Southern Medical University Shenzhen 518000 China qiminsmu@126.com

## Abstract

The treatment of malignant melanoma, an aggressive skin cancer with high recurrence and metastatic potential, often presents challenges due to systemic toxicity and multidrug resistance in current therapies. In this study, we develop a localized therapeutic platform by integrating methotrexate–manganese (MTX–Mn) coordination nanoparticles into a thermosensitive PLGA–PEG–PLGA (PPP) hydrogel to achieve sustained and site-specific drug delivery for melanoma treatment. MTX–Mn nanoparticles were synthesized through coordination-driven self-assembly and comprehensively characterized by FTIR, ^1^H NMR, TEM, EDS, and zeta potential analyses, confirming successful coordination and uniform nanoscale features. *In vitro* experiments demonstrated that MTX–Mn significantly inhibited melanoma cell proliferation and migration. Flow cytometry further revealed that MTX–Mn induced pronounced G0/G1 cell-cycle arrest and effectively promoted apoptosis, which was confirmed by TUNEL staining. Mechanistic investigations indicated that MTX–Mn triggered severe mitochondrial dysfunction, including ultrastructural damage, elevated mitochondrial ROS generation, and collapse of mitochondrial membrane potential (JC-1), suggesting a mitochondria-associated antitumor mechanism. *In vivo*, peritumoral administration of PPP/MTX–Mn markedly suppressed tumor growth in a melanoma-bearing mouse model, reduced the tumor burden, and significantly decreased Ki-67 expression. Histological evaluation of major organs by H&E staining revealed no obvious pathological abnormalities, supporting good *in vivo* biosafety. Overall, this MTX–Mn-loaded thermosensitive hydrogel provides a promising strategy for effective and safe localized melanoma therapy.

## Abstract 2

An injectable thermosensitive hydrogel delivering MTX–Mn nanoparticles enables sustained release, induces ROS-mediated apoptosis, and effectively suppresses melanoma growth.

## Graphical abstract

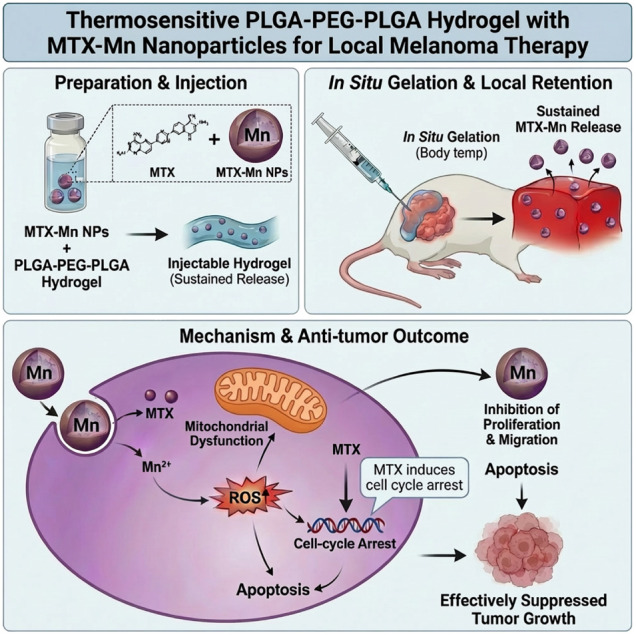

## 1 Introduction

Malignant melanoma is the most prevalent form of malignant skin cancer worldwide, representing a major threat to human health, and has emerged as a critical area of research in oncology.^1,2^ Traditional clinical treatments, such as surgery, chemotherapy, and radiotherapy, often have limitations and are accompanied by severe side effects. For example, surgical resection may fail to completely remove tumor tissue, leading to tumor residue, which can cause recurrence or metastasis; additionally, surgery may require further reconstructive procedures to repair tissue defects.^3–5^ Chemotherapy, on the other hand, may induce systemic toxicity, leading to symptoms such as hair loss, nausea, vomiting, and cardiovascular adverse effects.^6–8^ These challenges have driven the search for innovative therapeutic approaches.

In the current treatment of melanoma, multidrug resistance (MDR) to traditional drugs leads to tumor recurrence and metastasis, severely reducing the effectiveness of cancer therapies.^9^ This has become the main reason for the failure of clinical chemotherapy.^10^ Therefore, overcoming resistance is crucial for melanoma treatment. The use of novel drug combinations has emerged as an effective strategy to enhance therapeutic efficacy.^11,12^ Nanomedicine holds tremendous potential in the treatment of cancers, including melanoma.^13,14^ Among various nanotherapeutic strategies, metal-drug coordination complexes have attracted significant attention due to their unique physicochemical and biological properties.^15–17^ Methotrexate (MTX), a classical antifolate chemotherapeutic agent, inhibits dihydrofolate reductase and suppresses DNA synthesis, thereby exerting antitumor effects.^18,19^ Conversely, manganese ions play a crucial role in redox reactions and tumor progression, and manganese-based therapies have been shown to induce oxidative stress, disrupt mitochondrial function, and trigger cancer cell apoptosis.^20–23^ The coordination of MTX with manganese combines the antifolate activity with manganese-mediated cytotoxic mechanisms, resulting in a synergistic antitumor effect.

Despite these advantages, the effective and localized delivery of MTX–Mn nanotherapeutics to tumor tissues remains a critical challenge. Therefore, developing a suitable carrier system to achieve site-specific, sustained delivery is essential for fully exploiting the therapeutic potential of MTX–Mn-based nanomedicine. In this context, the development of multifunctional drug delivery systems (DDSs) has become a key strategy for improving melanoma treatment. Polymer-based hydrogels, particularly thermosensitive hydrogels, have garnered increasing interest for their ability to deliver drugs in a controlled manner and promote localized therapy. Thermosensitive hydrogels, such as poly(lactic-*co*-glycolic acid)–poly(ethylene glycol)–poly(lactic-*co*-glycolic acid) (PLGA–PEG–PLGA), undergo a reversible sol-to-gel transition in response to temperature changes, making them ideal for minimally invasive delivery. These hydrogels are liquid at low temperatures, allowing for easy injection, and then transition to a gel at physiological temperatures, enabling sustained drug release at the site of action.^24–28^ Such hydrogels provide controlled drug release and offer advantages in terms of biocompatibility and tissue regeneration.

**Fig. 1:**
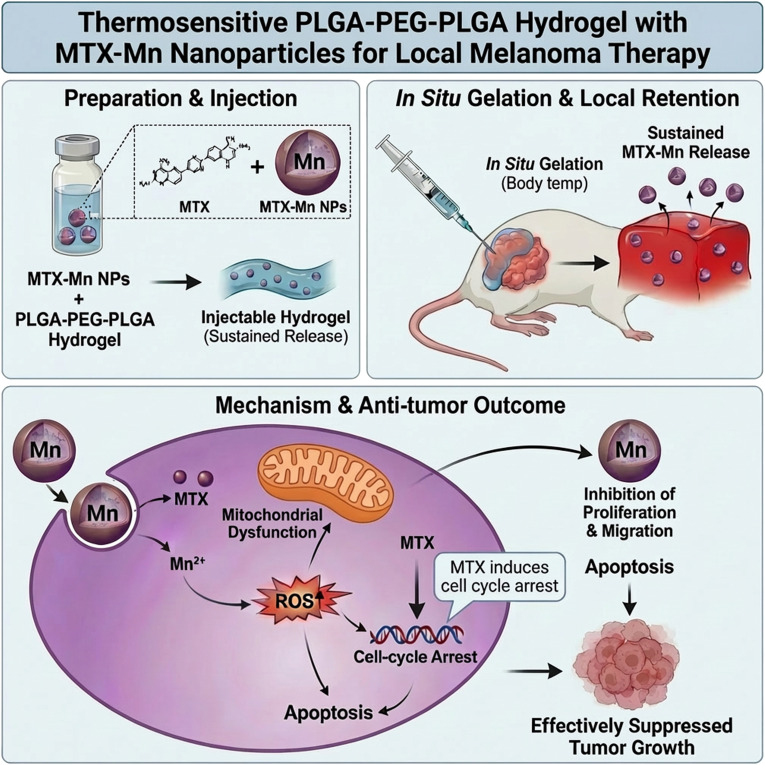
Thermosensitive PLGA–PEG–PLGA hydrogel loaded with MTX–Mn nanoparticles for localized melanoma therapy. Methotrexate–manganese (MTX–Mn) coordination nanoparticles are incorporated into a thermosensitive PLGA–PEG–PLGA hydrogel to form an injectable drug delivery system. After peritumoral injection, the hydrogel undergoes *in situ* gelation at body temperature, enabling prolonged local retention and sustained release of MTX–Mn at the tumor site. Released MTX–Mn induces excessive reactive oxygen species (ROS) generation and mitochondrial dysfunction, leading to cell-cycle arrest and apoptosis, thereby inhibiting tumor cell proliferation and migration and effectively suppressing melanoma growth.

In this study, we developed an innovative drug delivery system by incorporating MTX–Mn coordination nanoparticles into thermosensitive PLGA–PEG–PLGA hydrogels for localized melanoma treatment (Fig. 1). The thermosensitive hydrogel matrix enables controlled release of MTX–Mn nanoparticles, thereby enhancing therapeutic efficacy while minimizing systemic toxicity. The effectiveness of this functional system was evaluated through both *in vitro* and *in vivo* studies, demonstrating its potential as a novel and promising approach for melanoma therapy.

## 2 Materials and methods

### 2.1. Materials

Methotrexate (MW = 454.44 Da) was obtained from Macklin (China). Manganese(ii) chloride was purchased from Aladdin Reagent Co., Ltd (China). PLGA (1500–2000)–PEG (1000–1500)–PLGA (1500–2000) thermosensitive hydrogel was purchased from Shanghai Yuanye Bio-Technology Co., Ltd (Shanghai, China). All materials and reagents were used as received without further purification unless otherwise stated. Deionized (DI) water with a resistivity of >18.2 MΩ cm was used throughout the study. The essential reagents for cell culture included 1% penicillin–streptomycin solution (PS), fetal bovine serum (FBS), trypsin–EDTA solution, and Dulbecco's Modified Eagle Medium (DMEM), all purchased from Gibco (New York, USA). The mouse melanoma cell line B16F10 was obtained from Servicebio Biotechnology Co., Ltd (Wuhan, China).

### 2.2. Synthesis and characterization of the MTX–Mn complex

Methotrexate (MTX) and manganese(ii) chloride (MnCl_2_) were separately dissolved in *N*,*N*-dimethylformamide (DMF). The two solutions were then mixed at a molar ratio of Mn^2+^ to MTX greater than 3 : 1 to ensure an excess of MnCl_2_ and thereby promote complete coordination of MTX. The reaction mixture was maintained at 50 °C under continuous magnetic stirring for 12 h. Subsequently, the reaction mixture was gradually added dropwise into deionized water under continuous stirring until microcrystals precipitated. The resulting MTX–Mn coordination product was collected by centrifugation at 4000 rpm for 5 min, washed twice with deionized water, and re-centrifuged to remove unreacted MnCl_2_ and excess DMF, and finally lyophilized to obtain the MTX–Mn powder.

Methotrexate (MTX) and MTX–Mn were characterized by Fourier transform infrared spectroscopy (FTIR) (PerkinElmer Frontier) over a wavenumber range of 500–4000 cm^−1^ at room temperature. ^1^H nuclear magnetic resonance (^1^H NMR) analysis of MTX and MTX–Mn was performed using a Bruker AVANCE III 500 MHz spectrometer equipped with an N_2_ cryo-platform Prodigy probe. The samples were dissolved in deuterated dimethyl sulfoxide (DMSO-d_6_), and the measurements were conducted at room temperature. The morphology of the MTX–Mn coordination particles was observed using transmission electron microscopy (TEM), and the surface elemental composition of MTX–Mn was analyzed by energy-dispersive X-ray spectroscopy (EDS) (EDAX Octane Pro). The manganese content in MTX–Mn was quantified using inductively coupled plasma mass spectrometry (ICP-MS) (Agilent 7700X). The particle size and zeta potential of the MTX–Mn nanoparticles were characterized using a Zetasizer Nano ZS (Malvern Instruments, UK). The chemical stability of MTX–Mn was assessed *via* high-performance liquid chromatography (HPLC) (Agilent 1260 Infinity) with a diode array detector, through determination of its content and purity on day 0 and after seven days.

### 2.3. Cell proliferation

B16F10 cells were maintained in Dulbecco's Modified Eagle Medium (DMEM) supplemented with 10% fetal bovine serum (FBS), 50 U per mL penicillin G, and 50 µg per mL streptomycin sulfate. Cells were seeded into 96-well plates at a density of 1 × 10^3^ cells per well and cultured at 37 °C in a humidified incubator with 5% CO_2_. After 12 h of attachment, the cells were treated with various drugs, and cell proliferation was evaluated using a Cell Counting Kit-8 (CCK-8) assay (Beyotime, China) on days 0, 1, 3, and 5.

### 2.4. Cell apoptosis and cycle detection

Cell apoptosis was analyzed using an Annexin V Alexa Fluor 647/PI Apoptosis Assay Kit (4A Biotech, China) according to the manufacturer's instructions. Briefly, cells were treated with different concentrations of MTX–Mn for 48 h, harvested, washed with ice-cold phosphate-buffered saline (PBS), and resuspended in 100 µL of binding buffer. Subsequently, the cells were incubated with 5 µL of Annexin V Alexa Fluor 647 solution at room temperature for 5 min. Afterwards, 10 µL of propidium iodide (PI, 20 µg mL^−1^) and 400 µL of PBS were added. The stained cells were immediately analyzed by bivariate flow cytometry using a FACSCanto II (BD Accuri™ C6 FlowCytometer). Cells from different drug treatment groups were collected and washed once with PBS, followed by fixation with pre-cooled 75% ethanol at −20 °C overnight. After fixation, the cells were centrifuged and washed with PBS, and then incubated with PI staining buffer containing 0.1% NP-40, 10 µg per mL RNase A (Takara, Japan), and PI (1 : 500 dilution; Invitrogen, USA) in PBS for 30 min in the dark. Cell cycle distribution was analyzed by flow cytometry (BD Accuri™ C6) using the PE-A channel, and the data were processed using ModFit LT software.

### 2.5. TUNEL assay

Cell apoptosis was assessed using a TUNEL apoptosis detection kit (Wuhan, China) according to the manufacturer's instructions. Briefly, cells from different treatment groups were washed once with phosphate-buffered saline (PBS) and fixed with 4% paraformaldehyde (in PBS) at room temperature for 20 min. After fixation, the cells were washed twice with PBS and permeabilized with 0.2% Triton X-100 (in PBS) for 20 min at room temperature. Following two additional washes with PBS, the TUNEL reaction was carried out according to the kit protocol. Finally, TUNEL-positive cells were observed and quantified using a confocal laser scanning microscope.

### 2.6. Cell migration assay

To further evaluate the migratory ability of B16F10 cells, the cells were treated with different drugs for 72 h and then seeded at a density of 1 × 10^4^ cells per well into the upper chamber of a Transwell insert (8 µm pore size). The upper chamber was filled with serum-free medium, while the lower chamber contained 600 µL of complete medium supplemented with 10% fetal bovine serum. After incubation for 8 h, non-migrated cells on the upper surface of the membrane were gently removed with a cotton swab. The Transwell inserts were then fixed with 4% formaldehyde for 15 min and stained with 0.1% crystal violet for 20 min. After washing three times with PBS, images were captured and recorded. The number of cells that migrated to the underside of the membrane was quantified using ImageJ software.

### 2.7. Mitochondrial ultrastructure analysis by transmission electron microscopy

Mitochondrial ultrastructural changes were examined using transmission electron microscopy (TEM), which was performed by Servicebio Biotechnology Co., Ltd Following treatment with the different drugs for 3 days, B16F10 cells were harvested and immediately immersed in a TEM-specific fixative solution (Servicebio, G1102) at 4 °C for overnight fixation. The samples were subsequently post-fixed with 1% osmium tetroxide for 2 h under light-protected conditions. After fixation, the cell pellets were sequentially dehydrated in increasing concentrations of ethanol and embedded in EMbed 812 resin (SPI, 90529-77-4). Ultrathin sections with a thickness of approximately 60 nm were cut using an ultramicrotome (Leica, UC7). The sections were contrast-enhanced by staining with 2% uranyl acetate in absolute ethanol for 8 min, followed by 2.6% lead citrate for an additional 8 min. Imaging and ultrastructural evaluation were carried out using a Hitachi HT7700 transmission electron microscope.

### 2.8. Mitochondrial superoxide analysis

Intramitochondrial superoxide generation was assessed using the fluorescent probe MitoSOX Red (Molecular Probes, M36008) in accordance with the manufacturer's protocol. Briefly, B16F10 cells subjected to different treatments were rinsed with Hank's balanced salt solution (HBSS) supplemented with Ca^2+^ and Mg^2+^, and then incubated with MitoSOX Red at 37 °C for 30 min in the absence of light. Following incubation, the nuclei were counterstained with Hoechst dye, and fluorescent signals were visualized using a confocal laser scanning microscope. For quantitative analysis, MitoSOX Red-labeled cells were detached by trypsinization, washed again with HBSS, and analyzed by flow cytometry to determine the relative mitochondrial superoxide production. For mitochondrial membrane potential analysis, changes in mitochondrial membrane potential (Δ*Ψ*_m_) were evaluated using the JC-1 fluorescent dye (Beyotime, C2006). Treated cells were collected, washed with HBSS containing Ca^2+^ and Mg^2+^, and subsequently incubated with JC-1 dye at 37 °C for 20 min. After staining, the cells were further incubated with Hoechst dye for 10 min to label the nuclei. The fluorescence distribution of JC-1 was examined by confocal laser scanning microscopy to assess alterations in mitochondrial membrane potential.

### 2.9. Synthesis of the PLGA–PEG–PLGA/MTX–Mn (PPP/MTX–Mn) hydrogel

The hydrogel was prepared as previously reported.^29–31^ Briefly, PLGA (1500–2000)–PEG (1000–1500)–PLGA (1500–2000) thermosensitive copolymer was gradually added to deionized water under continuous stirring to obtain hydrogels at different concentrations (5%, 10%, and 20%, w/v). The mixtures were incubated at 4 °C for 7 days, during which the copolymer was completely dissolved, yielding clear and homogeneous viscous solutions. Subsequently, a predetermined amount of MTX–Mn was added to the hydrogel solutions, followed by overnight stirring at 4 °C to ensure uniform dispersion of MTX–Mn within the hydrogel matrix.

### 2.10. Mechanical properties evaluation of the hydrogel

The mechanical properties of the PPP/MTX–Mn hydrogel were strongly influenced by both polymer concentration and temperature. To systematically investigate these effects, the gelation behavior and elastic modulus of the PPP/MTX–Mn hydrogels were evaluated at different concentrations ranging from 5% to 20% (w/v). Rheological measurements were performed using a rotational rheometer equipped with a parallel-plate geometry (Thermo Fisher Scientific, HAAKE MARS 40), as previously described.^32,33^ Hydrogel samples at concentrations of 5%, 10%, and 20% (w/v) were prepared according to the procedures described above and carefully placed onto the rheometer stage. Temperature-dependent rheological tests were then conducted to assess the flow behavior and gelation characteristics of the hydrogels. Dynamic oscillatory rheological analyses were further carried out to characterize the viscoelastic properties of the hydrogels. Time sweep measurements were performed at a constant oscillation frequency and strain within the linear viscoelastic region, while gradually varying the temperature, to monitor changes in the storage modulus (*G*′) and loss modulus (*G*″). The resulting *G*′ and *G*″ values of the hydrogels with different concentrations were recorded and compared to evaluate their elastic and viscous behaviors under varying thermal conditions.

### 2.11. Characterization of the hydrogel

The microstructural morphology of the PPP/MTX–Mn hydrogel was investigated using scanning electron microscopy (SEM) at an accelerating voltage of 10 kV to elucidate its internal and surface structural characteristics. In addition, the elemental composition and spatial distribution on the hydrogel surface were analyzed by energy-dispersive X-ray spectroscopy (EDS). Prior to SEM observation, the hydrogel sample was freeze-dried and sputter-coated with a thin layer of platinum to improve surface conductivity and obtain high-resolution images.

### 2.12. *In vitro* release of MTX–Mn quantified by HPLC

The *in vitro* release profile of MTX–Mn from the PPP/MTX–Mn hydrogel was evaluated in physiological saline at 37 °C to simulate physiological conditions. Briefly, 100 mg of hydrogel was placed in 50 mL of physiological saline and incubated at 37 °C with gentle agitation to assess its sustained drug release behavior. At designated time intervals, 1 mL of the release medium was collected and immediately replenished with an equal volume of fresh saline to maintain sink conditions. The amount of MTX–Mn released into the medium was determined by high-performance liquid chromatography (HPLC) using an external standard calibration curve generated from MTX–Mn reference solutions. The cumulative release of MTX–Mn was calculated as the percentage of the total amount released at each time point relative to the initial drug loading within the hydrogel.

### 2.13. *In vivo* antitumor efficacy

Male C57BL/6 mice (6–8 weeks old) were purchased from Guangdong Yaokang Biotechnology Co., Ltd, Jiangsu Province, China. To establish melanoma-bearing mouse models, B16F10 cells were collected and washed twice with PBS. The cells were then resuspended in pre-chilled 50% Matrigel diluted in PBS on ice. A total of 1 × 10^6^ cells were injected subcutaneously into the flank of each mouse. When the tumor volumes reached approximately 100 mm^3^, the mice were randomly divided into four groups (*n* = 4 per group), namely the PLGA–PEG–PLGA hydrogel (PPP, control group), PLGA–PEG–PLGA hydrogel/manganese (PPP/Mn), PLGA–PEG–PLGA hydrogel/methotrexate (PPP/MTX), and PLGA–PEG–PLGA hydrogel/methotrexate–manganese (PPP/MTX–Mn) groups. Each group received the corresponding formulation *via* peritumoral injection, with an injection volume of 25 µL per treatment. Treatment with PPP/MTX–Mn hydrogel was initiated accordingly. At the end of the experiment, the mice were sacrificed by cervical dislocation, and the tumors as well as the major organs (heart, liver, spleen, lungs, and kidneys) were immediately excised for further analysis.

### 2.14. Immunohistochemistry (IHC) staining

Immunohistochemical staining was performed to evaluate tumor cell proliferation using Ki-67 as a marker. Briefly, paraffin-embedded tumor tissues were sectioned into 4–5 µm slices, deparaffinized, and rehydrated. Antigen retrieval was carried out using citrate buffer (pH 6.0) or EDTA buffer (pH 8.0–9.0) with heat-mediated treatment. After cooling to room temperature, sections were washed with PBS and incubated with 3% hydrogen peroxide for 10 min to block endogenous peroxidase activity. Non-specific binding was blocked with 5% bovine serum albumin (BSA) or normal serum for 30–60 min at room temperature. The sections were then incubated with the primary antibody against Ki-67 (1 : 1000, Proteintech) at 4 °C overnight. After washing, the sections were incubated with an HRP-conjugated secondary antibody for 30–60 min at room temperature. Color development was achieved using DAB substrate, followed by counterstaining with hematoxylin. Finally, the slides were dehydrated, cleared, and mounted. Images were captured under a microscope, and Ki-67-positive cells were quantified using ImageJ.

### 2.15. Hematoxylin and eosin (HE) staining

The major organs (heart, liver, spleen, lungs, and kidneys) were harvested from the mice, rinsed with saline to remove residual blood, and fixed in 4% paraformaldehyde for 24 h. The samples were then dehydrated through a graded ethanol series, cleared in xylene, and embedded in paraffin. The paraffin blocks were sectioned into 4–5 µm slices, followed by deparaffinization and rehydration. The sections were stained with hematoxylin for 3–5 min, rinsed under running water, differentiated in 1% acid alcohol, and blued in tap water. Subsequently, the sections were counterstained with eosin for 1–3 min. After staining, the slides were dehydrated, cleared in xylene, and mounted with neutral resin. Histological morphology was observed and imaged using an optical microscope.

### 2.16. Statistical analysis

To ensure the reliability and reproducibility of the data, all experiments were performed independently in triplicate. Statistical analyses were conducted using GraphPad Prism 9 (GraphPad, USA) and Origin 2021 (OriginLab, USA). Data are presented as mean ± standard deviation (SD). Comparisons between two groups were performed using Student's *t*-test. A *P* value < 0.05 was considered statistically significant. Statistical significance is indicated as follows: *P* < 0.05 (*), *P* < 0.01 (**), *P* < 0.001 (***), and *P* < 0.0001 (****).

## 3 Results and discussion

### 3.1. Synthesis and characterization of MTX–Mn coordination nanoparticles

**Fig. 2:**
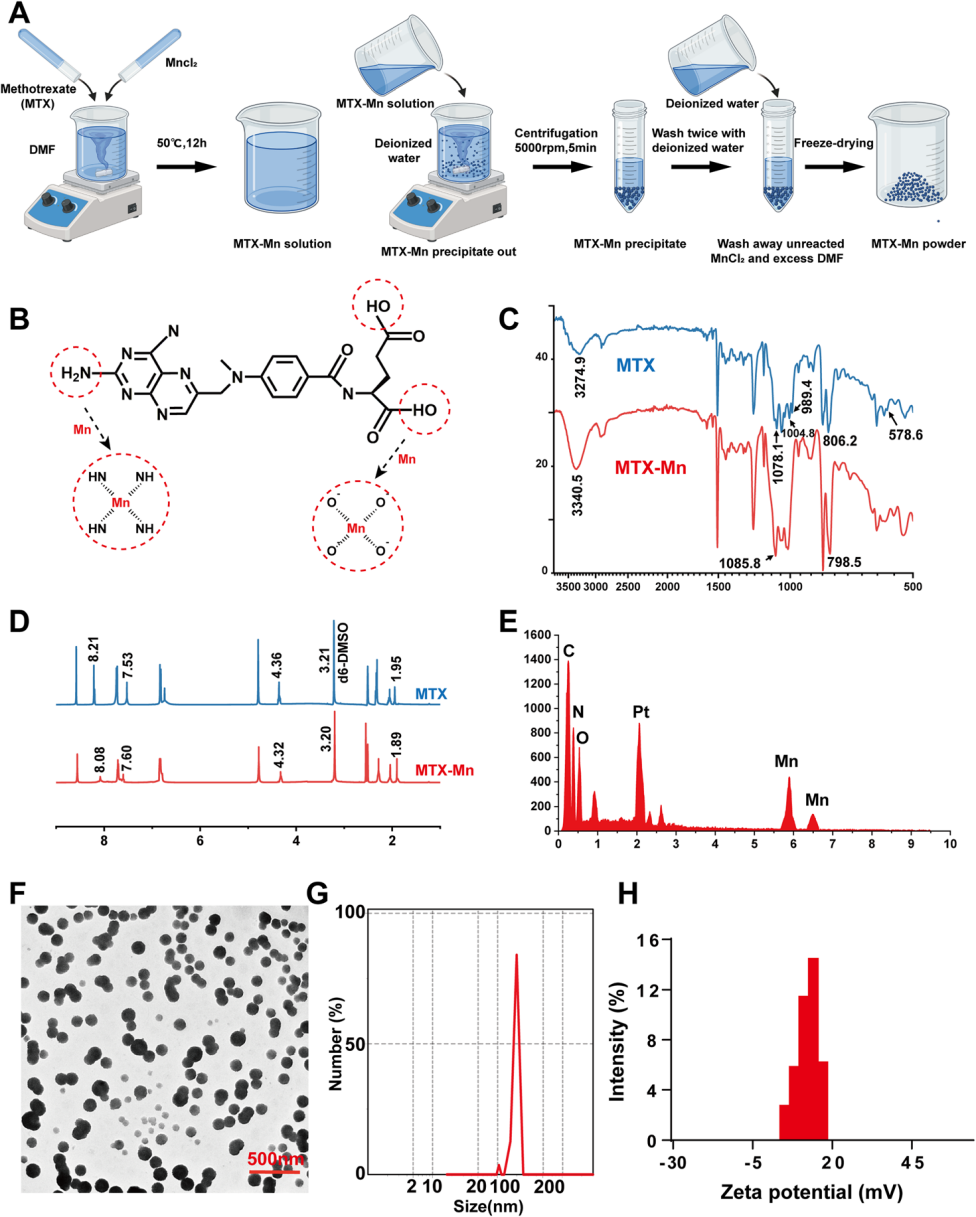
Synthesis and characterization of the MTX–Mn coordination nanoparticles. (A) Schematic illustration of the synthesis procedure of the MTX–Mn coordination nanoparticles. (B) Proposed coordination mechanism between MTX and Mn^2+^ ions. (C) FTIR spectra of MTX and MTX–Mn. (D) ^1^H NMR spectra of MTX and MTX–Mn. (E) Energy-dispersive X-ray spectroscopy (EDS) analysis confirming the presence of Mn in the MTX–Mn coordination nanoparticles. (F) Transmission electron microscopy (TEM) image of the MTX–Mn nanoparticles. (G) The particle size distribution of the MTX–Mn nanoparticles. (H) Zeta potential distribution of the MTX–Mn nanoparticles, indicating favorable surface charge and colloidal stability.

Many drugs are rich in metal coordination groups, such as amino, carboxyl, and heterocyclic oxygen and nitrogen. They can form coordination complexes with various metal ions, yielding nanoparticles (NPs).^34^ These coordination products can integrate the advantages of both components, thereby exerting synergistic therapeutic effects.^35,36^ As illustrated in Fig. 2A, methotrexate (MTX) was successfully coordinated with Mn^2+^ ions to form MTX–Mn complexes *via* a coordination-driven self-assembly process. After purification and freeze-drying, MTX–Mn coordination products were obtained as a stable powder. The coordination mechanism between MTX and Mn^2+^ is schematically proposed in Fig. 2B. Mn^2+^ ions are primarily coordinated with the nitrogen atoms of the pteridine ring and the oxygen atoms of the carboxyl groups in MTX, forming stable metal–ligand coordination bonds.

Fourier transform infrared (FTIR) spectroscopy was employed to confirm the coordination interaction between MTX and Mn^2+^ (Fig. 2C). Compared with free MTX, the MTX–Mn complexes exhibited pronounced shifts and intensity variations in several characteristic absorption bands. For free MTX, the broad absorption band at approximately 3275 cm^−1^, corresponding to –NH/–OH stretching vibrations, shifted to around 3340 cm^−1^ after coordination. In addition, the characteristic C=O stretching vibration of MTX observed at approximately 1600–1630 cm^−1^ showed noticeable changes in both intensity and peak position in the MTX–Mn spectrum. Furthermore, absorption bands in the fingerprint region, including peaks near 1078 cm^−1^ and 806 cm^−1^, were shifted to approximately 1086 cm^−1^ and 798 cm^−1^, respectively, after coordination. These spectral changes indicate that the amino and carboxyl groups of MTX participated in coordination with Mn^2+^ ions, confirming the successful formation of MTX–Mn coordination complexes.

^1^H NMR spectroscopy further supported the coordination between MTX and Mn^2+^ (Fig. 2D). In the spectrum of free MTX, characteristic proton signals were observed at approximately *δ* 8.21, 7.53, 4.36, 4.21, and 1.95 ppm. After coordination with Mn^2+^, these signals exhibited distinct chemical shift changes and peak broadening, with corresponding peaks appearing at approximately *δ* 8.08, 7.60, 4.32, 3.20, and 1.89 ppm in the MTX–Mn complexes. The observed peak broadening and chemical shift variations are attributed to changes in the electronic environment of MTX protons induced by Mn^2+^ coordination, further confirming the formation of MTX–Mn coordination structures. Elemental composition analysis using energy-dispersive X-ray spectroscopy (EDS) revealed the presence of Mn in the MTX–Mn complexes (Fig. 2E), further confirming the successful incorporation of Mn^2+^ into the coordination structure. Inductively coupled plasma mass spectrometry (ICP-MS) analysis revealed an average Mn^2+^-to-MTX mass ratio of 0.17 in the resulting MTX–Mn complex, further confirming the successful formation of the coordination structure (Table 1). The morphology of the MTX–Mn complexes was examined by transmission electron microscopy (TEM). As shown in Fig. 2F, the MTX–Mn complexes formed uniform and well-dispersed spherical nanoparticles with nanoscale dimensions. The particle size distribution of the MTX–Mn nanoparticles was characterized by dynamic light scattering (DLS) using a Malvern particle size analyzer, which revealed a narrow size distribution centered at approximately 100 nm (Fig. 2G), indicating good size uniformity.

**Table 1:** Mn^2+^/MTX ratio in MTX–Mn determined by ICP-MS

	MTX–Mn weight (mg)	MTX–Mn volume (L)	Mn^2+^ conc. (ppb)	Mn^2+^ mass (mg)	Mn^2+^/MTX (mg mg^−1^)
Sample1	15	10	214.933966	2.14934	0.167255223
Sample2	15	10	232.780434	2.32780	0.183693439
Sample3	15	10	220.401901	2.20402	0.172243158

The surface charge of the MTX–Mn nanoparticles was further evaluated by zeta potential measurements (Fig. 2H). The nanoparticles exhibited a positive zeta potential of approximately +10 to +15 mV, suggesting good colloidal stability and favorable surface properties for subsequent biomedical applications.

High-performance liquid chromatography (HPLC) results confirmed that MTX–Mn exhibited good chemical stability over a period of 7 days. The chromatographic profiles recorded on day 0 and day 7 were highly consistent, showing almost complete overlap. No new impurity peaks appeared, and no significant variation in absorbance was observed. These results suggest that MTX–Mn retained its structural integrity throughout storage and showed no evidence of measurable degradation within the seven-day period (Fig. S1A).

### 3.2. Antitumor activity of the MTX–Mn complexes *in vitro*

**Fig. 3:**
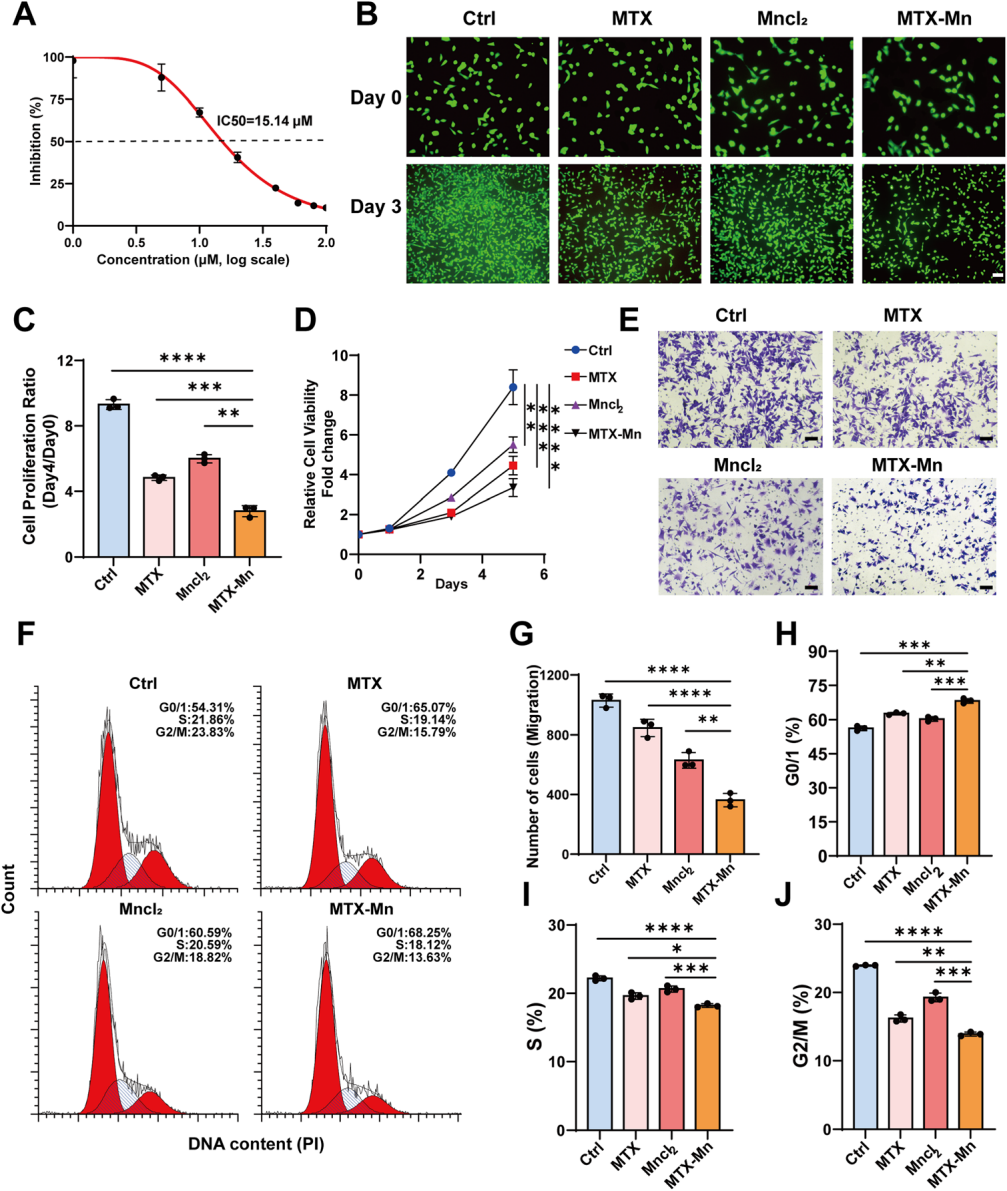
*In vitro* antitumor effects of MTX–Mn coordination nanoparticles on B16F10 melanoma cells. (A) Dose–response curve of MTX–Mn in B16F10 cells determined by CCK-8 assay, showing an IC_50_ value of approximately 15.14 µM. (B) Representative fluorescence images of B16F10 cells treated with different formulations at day 0 and day 3. (C) Quantitative analysis of cell proliferation ratio (day 3/day 0) under different treatments. (D) Cell proliferation curves of B16F10 cells over 5 days. (E) Representative Transwell migration images of B16F10 cells after treatment with different formulations. (F) Flow cytometry histograms showing cell-cycle distribution of B16F10 cells following different treatments. (G) Quantitative analysis of migrated cell numbers from the Transwell assay. (H–J) Quantitative analysis of cell-cycle distribution. Data are presented as mean ± SD (*n* = 3). Statistical significance is indicated as *P < 0.05, **P < 0.01, ***P < 0.001, and ****P < 0.0001.

The cytotoxicity of the MTX–Mn complexes was first evaluated using a dose–response assay. As shown in Fig. 3A, MTX–Mn exhibited a concentration-dependent inhibitory effect on B16F10 cell proliferation, with an IC_50_ value of approximately 15.14 µM, indicating potent antitumor activity. Fluorescence microscopy was further employed to visualize changes in fluorescence-labeled cell density following different treatments (Fig. 3B and C). Representative images acquired at day 0 and day 3 showed a substantial increase in cell density in the control group over time, whereas treatment with MTX or MnCl_2_ moderately suppressed cell proliferation. In contrast, MTX–Mn treatment resulted in a marked reduction in cell density at day 3 compared with the control and single-agent groups, demonstrating a stronger antiproliferative effect. Consistently, CCK-8 analysis revealed that MTX–Mn induced a pronounced and time-dependent decrease in cell viability relative to MTX or MnCl_2_ alone (Fig. 3D), further confirming the enhanced growth–inhibitory activity of the coordination complexes.

In addition to suppressing proliferation, the anti-migratory effect of MTX–Mn was investigated using a Transwell migration assay. Representative images (Fig. 3E) and quantitative analysis (Fig. 3G) showed that MTX–Mn significantly reduced the number of migrated B16F10 cells compared with the control, MTX, and MnCl_2_ groups, indicating effective inhibition of tumor cell migration. To explore the potential mechanism, cell cycle distribution was analyzed by flow cytometry (Fig. 3F). MTX–Mn treatment induced marked cell cycle arrest at the G0/G1 phase, accompanied by reductions in the S and G2/M phases. Quantitative results further confirmed a significant increase in the G0/G1 population (Fig. 3H) and corresponding decreases in the S (Fig. 3I) and G2/M (Fig. 3J) phases compared with the other treatment groups.

Importantly, the biosafety of MTX–Mn toward normal cells was evaluated using NIH/3T3 fibroblasts treated with 15.14 µM MTX–Mn. Annexin V-FITC/PI flow cytometry analysis showed that although a slightly higher apoptotic cell population was observed in the MTX–Mn-treated group compared with the control, the difference was not statistically significant. These results indicate that MTX–Mn does not induce obvious apoptosis in normal fibroblasts at the tested concentration (Fig. S1B and C). In contrast, 15.14 µM MTX–Mn exhibited pronounced pro-apoptotic effects on melanoma cells. TUNEL staining demonstrated a markedly increased number of TUNEL-positive B16F10 cells following MTX–Mn treatment compared with the ctrl, MTX, and MnCl_2_ groups (Fig. S1D and E).

Collectively, these results demonstrate that MTX–Mn coordination complexes exhibit enhanced *in vitro* antitumor activity by inhibiting tumor cell proliferation and migration, inducing G0/G1 cell cycle arrest, and promoting apoptosis in melanoma cells, outperforming free MTX or MnCl_2_ alone.

### 3.3. MTX–Mn induces mitochondrial dysfunction and ROS generation in B16F10 cells

**Fig. 4:**
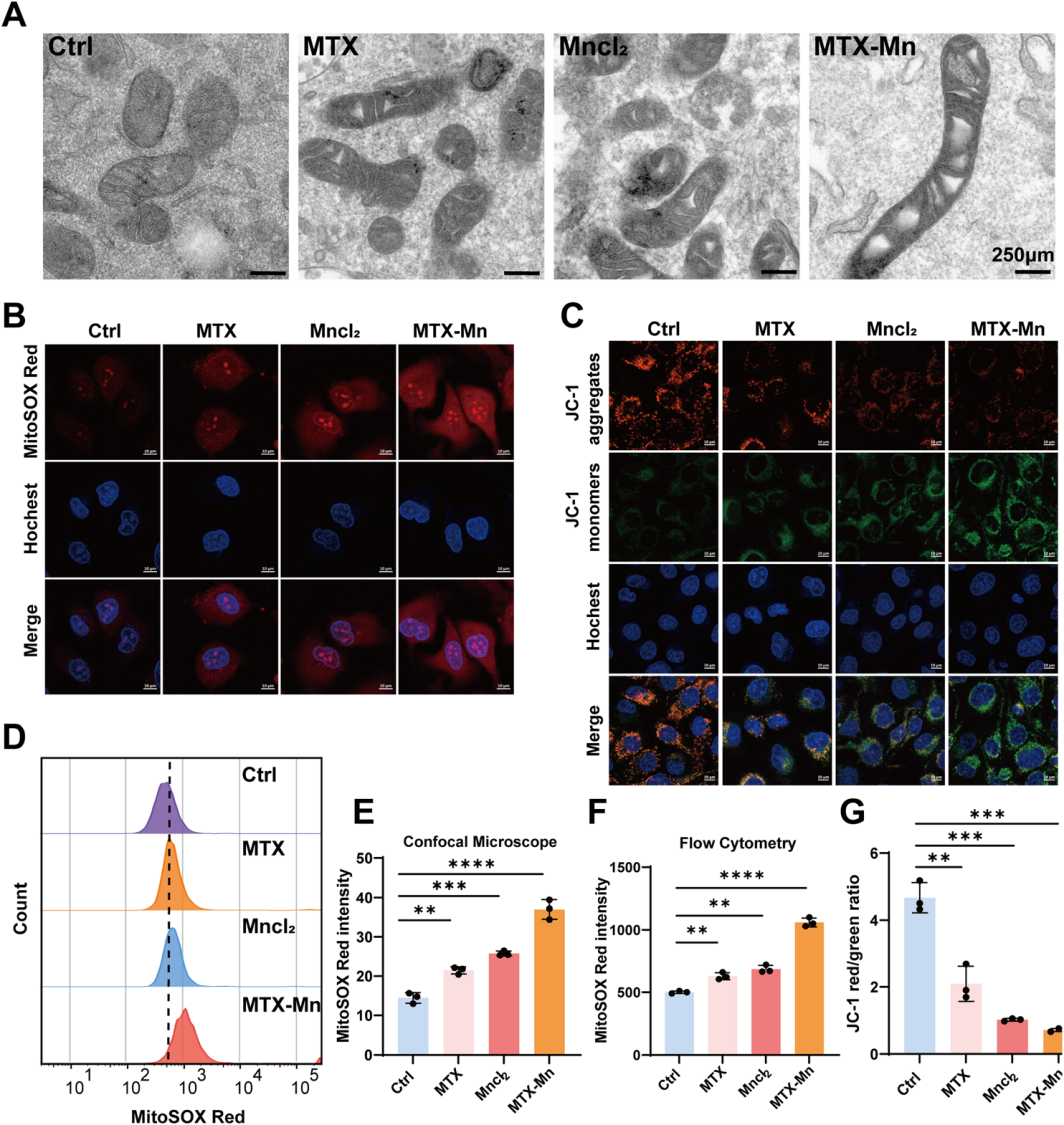
MTX–Mn induces mitochondrial dysfunction and oxidative stress in B16F10 melanoma cells. (A) Transmission electron microscopy (TEM) images showing mitochondrial ultrastructural changes in B16F10 cells. (B) Confocal laser scanning microscopy images of mitochondrial superoxide production detected by MitoSOX Red staining. (C) Confocal fluorescence images of mitochondrial membrane potential assessed by JC-1 staining. (D) Representative flow cytometry histograms of MitoSOX Red fluorescence intensity in B16F10 cells under different treatments. (E) Quantitative analysis of mitochondrial ROS levels based on confocal microscopy images. (F) Quantitative analysis of MitoSOX Red fluorescence intensity measured by flow cytometry. (G) Quantification of mitochondrial membrane potential expressed as the JC-1 red/green fluorescence ratio. Data are presented as mean ± SD (*n* = 3). Statistical significance is indicated as **P < 0.01, ***P < 0.001, and ****P < 0.0001.

Manganese exposure has been demonstrated to disrupt mitochondrial function and promote the generation of reactive oxygen species (ROS). Mechanistically, manganese's redox activity facilitates ROS production and oxidative stress, leading to mitochondrial dysfunction, membrane potential loss, and activation of cell death pathways.^37–41^ Several studies have reported incorporating MnCl_2_ or Mn–drug coordination complexes into hydrogels to achieve sustained release for antitumor therapy.^42,43^ In this study, mitochondrial ultrastructural changes were examined by transmission electron microscopy (TEM). As shown in Fig. 4A, mitochondria in the control group exhibited an intact morphology with well-defined cristae. In contrast, treatment with MTX or MnCl_2_ induced mitochondrial swelling and partial disruption of the cristae structure. Notably, MTX–Mn treatment resulted in severe mitochondrial structural alterations, characterized by pronounced mitochondrial swelling and a marked loss of cristae integrity, indicating significant mitochondrial dysfunction.

To further evaluate mitochondrial reactive oxygen species (ROS) generation, B16F10 cells were stained with MitoSOX Red and observed by confocal laser scanning microscopy (Fig. 4B). Compared with the control group, MTX or MnCl_2_ treatment led to a moderate increase in red fluorescence intensity, whereas MTX–Mn treatment induced a marked enhancement of MitoSOX Red fluorescence, indicating a substantial increase in mitochondrial ROS levels. Quantitative analysis of fluorescence intensity obtained from confocal images confirmed that MTX–Mn significantly elevated mitochondrial ROS production compared with single-agent treatments (Fig. 4E). Consistently, flow cytometry analysis further validated the pronounced increase in MitoSOX Red intensity in the MTX–Mn group (Fig. 4D and F).

Mitochondrial membrane potential (Δ*Ψ*_m_) was subsequently assessed using JC-1 staining. As shown in Fig. 4C, control cells exhibited strong red fluorescence corresponding to JC-1 aggregates, indicating intact mitochondrial membrane potential. In contrast, MTX or MnCl_2_ treatment caused a partial loss of red fluorescence accompanied by increased green fluorescence, suggesting mitochondrial depolarization. Notably, MTX–Mn treatment resulted in a pronounced shift from red to green fluorescence, indicating severe mitochondrial membrane potential collapse. Quantitative analysis of the JC-1 red/green fluorescence ratio further confirmed a significant reduction in Δ*Ψ*_m_ in the MTX–Mn group compared with the control and single-agent groups (Fig. 4G).

Collectively, these results demonstrate that the MTX–Mn coordination complexes induce excessive mitochondrial ROS generation and severe mitochondrial dysfunction, including loss of mitochondrial membrane potential, which may contribute to their enhanced antitumor activity. Notably, mitochondrial ROS were specifically assessed using MitoSOX to reflect mitochondrial oxidative stress. Combined with the mitochondrial ultrastructural damage observed by TEM and the loss of mitochondrial membrane potential revealed by JC-1 staining, these findings indicate pronounced mitochondrial oxidative stress. Nevertheless, the measurement of total intracellular ROS warrants further investigation in future studies.

### 3.4. Preparation and characterization of an MTX–Mn-loaded PLGA–PEG–PLGA thermosensitive hydrogel

The PLGA–PEG–PLGA hydrogel is a temperature-responsive triblock copolymer consisting of a hydrophilic A block, polyethylene glycol (PEG), and hydrophobic B blocks, poly(lactide-*co*-glycolide) (PLGA). At low temperatures, the copolymer is highly soluble in aqueous media and exists as a free-flowing solution. As the temperature increases above the sol–gel transition point, strengthened hydrophobic interactions among the PLGA segments drive the physical association of the polymer chains, leading to the formation of a three-dimensional hydrogel network without the need for chemical crosslinkers. This thermally induced sol–gel transition imparts excellent injectability to the system and enables *in situ* gelation under physiological conditions.^44^ Owing to these characteristics, temperature-sensitive PLGA–PEG–PLGA triblock hydrogels are well suited for sustained and localized drug delivery, allowing controlled release of therapeutic agents over several days.^45,46^

**Fig. 5:**
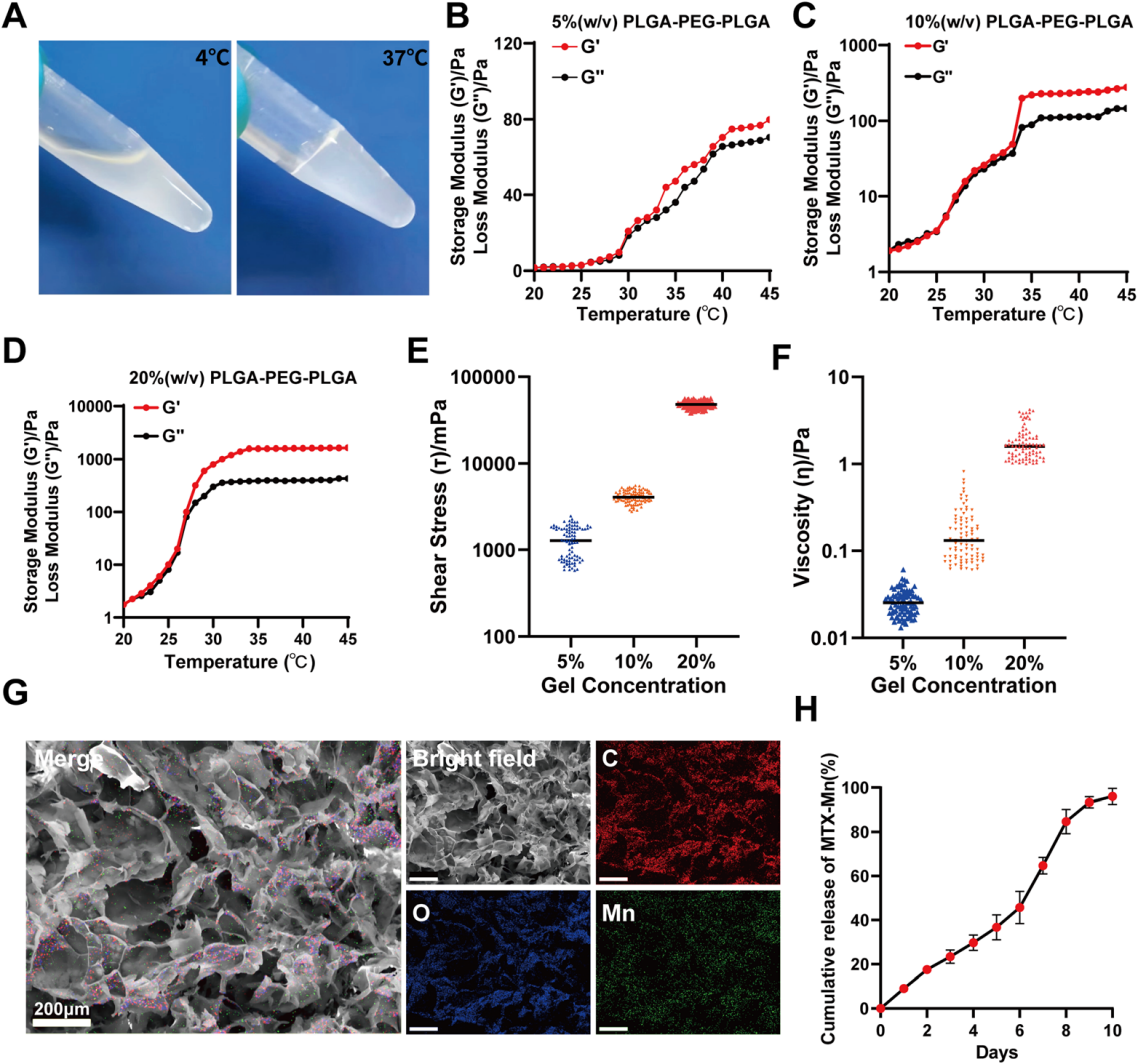
Thermosensitive behavior, rheological properties, microstructure, and drug release of the PLGA–PEG–PLGA hydrogels. (A) Photographs of the PLGA–PEG–PLGA hydrogel at 4 °C and 37 °C, showing temperature-induced sol–gel transition behavior. (B–D) Temperature-dependent rheological properties of the PLGA–PEG–PLGA hydrogels at different concentrations. (E) Shear stress of the PLGA–PEG–PLGA hydrogels at different concentrations. (F) Viscosity of the PLGA–PEG–PLGA hydrogels as a function of gel concentration. (G) Scanning electron microscopy (SEM) images of the MTX–Mn-loaded PLGA–PEG–PLGA hydrogel showing a porous three-dimensional microstructure, along with corresponding elemental mapping images of carbon (C), oxygen (O), and manganese (Mn). (H) *In vitro* cumulative release profile of MTX–Mn from the PLGA–PEG–PLGA hydrogel over time. Data are presented as mean ± SD (*n* = 3).

In this study, the sol–gel transition behavior of the PLGA–PEG–PLGA hydrogel was first evaluated. As shown in Fig. 5A, the PLGA–PEG–PLGA solution remained in a liquid state at 4 °C and rapidly transformed into a non-flowing gel at 37 °C, demonstrating its typical thermosensitive gelation behavior.

The temperature-dependent rheological properties of the PLGA–PEG–PLGA hydrogels at different polymer concentrations (5%, 10%, and 20%, w/v) were further analyzed. As shown in Fig. 5B–D, both the storage modulus (*G*′) and loss modulus (*G*″) increased with temperature, and a clear sol–gel transition occurred as *G*′ surpassed *G*″ near physiological temperature. Increasing the polymer concentration resulted in a marked enhancement of the mechanical properties of the hydrogel, with the 20% (w/v) hydrogel exhibiting the highest *G*′ values, indicating improved mechanical strength. This enhanced mechanical stability is crucial for maintaining the hydrogel at the injection site after local administration *in vivo*, thereby preventing undesired diffusion or migration from the injection region.

The injectability and mechanical robustness of the hydrogels were further evaluated by shear stress and viscosity measurements. As shown in Fig. 5E, the shear stress increased significantly with increasing polymer concentration, while viscosity measurements (Fig. 5F) revealed a concentration-dependent increase in viscosity.

The microstructure of the MTX–Mn-loaded hydrogel was characterized by scanning electron microscopy (SEM) combined with elemental mapping. As shown in Fig. 5G, the hydrogel exhibited a porous three-dimensional network structure. Energy-dispersive spectroscopy (EDS) mapping further confirmed the homogeneous distribution of carbon (C), oxygen (O), and manganese (Mn) elements throughout the hydrogel matrix, indicating the successful incorporation and uniform dispersion of MTX–Mn nanoparticles within the hydrogel.

The *in vitro* release behavior of MTX–Mn from the thermosensitive hydrogel was subsequently investigated. As shown in Fig. 5H, MTX–Mn was gradually released from the PLGA–PEG–PLGA hydrogel in a sustained manner over time, with cumulative release increasing continuously over 9 days, demonstrating the hydrogel's potential as a controlled drug delivery system.

### 3.5. *In vivo* antitumor efficacy and biosafety of the PPP/MTX–Mn hydrogel

Injectable hydrogel-based drug delivery systems have emerged as a promising strategy for tumor inhibition due to their ability to achieve localized and sustained therapeutic effects. Local injection allows direct drug administration at the tumor site, significantly enhancing intratumoral drug concentration while minimizing systemic exposure and adverse effects. Following injection, *in situ*-forming hydrogels create a drug-loaded depot that prolongs drug retention, enables controlled release, and maintains effective local drug levels.^47–53^

**Fig. 6:**
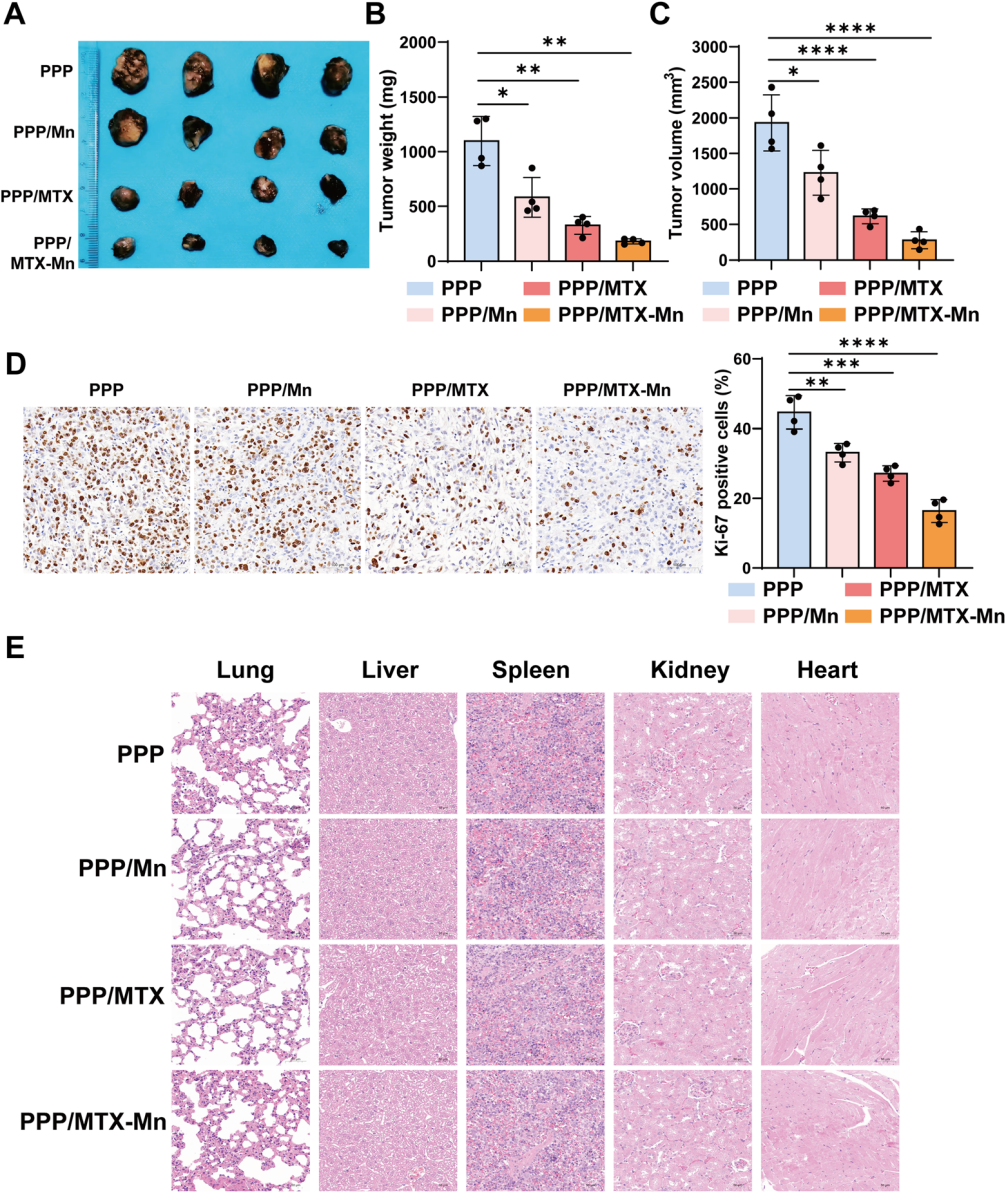
*In vivo* antitumor efficacy and biosafety of PPP/MTX–Mn hydrogel in a melanoma-bearing mouse model. (A) Representative photographs of excised tumors from mice treated with PPP, PPP/Mn, PPP/MTX, or PPP/MTX–Mn. (B) Quantitative analysis of tumor weight. (C) Quantitative analysis of tumor volume. (D) Representative immunohistochemical (IHC) staining images of Ki-67 in tumor tissues and corresponding quantitative analysis of Ki-67-positive cells. (E) Representative hematoxylin and eosin (HE) staining images of major organs from different treatment groups, showing no obvious histopathological abnormalities, indicating good *in vivo* biosafety of the hydrogel formulations. Data are presented as mean ± SD (*n* = 4). Statistical significance is indicated as *P < 0.05, **P < 0.01, ***P < 0.001, and ****P < 0.0001.

The *in vivo* antitumor efficacy of the PLGA–PEG–PLGA (PPP) hydrogel formulations was evaluated in a melanoma-bearing mouse model. To achieve a sustained antitumor effect, a PLGA–PEG–PLGA hydrogel loaded with MTX–Mn at a concentration of 151.4 µM (ten-fold higher than that used in *in vitro* experiments) was prepared. This concentration was determined based on the IC_50_ value obtained from *in vitro* experiments together with the *in vitro* sustained release profile of the hydrogel, ensuring effective antitumor activity and prolonged drug exposure *in vivo*. Based on the sustained-release properties of the PPP/MTX–Mn hydrogel, the hydrogel was administered once every 9 days. When the maximum tumor volume reached approximately 2500 mm^3^, mice in each group were sacrificed, and tissues were collected for analysis. As shown in Fig. 6A, the excised tumors from the PPP/MTX–Mn group were markedly smaller than those from the PPP, PPP/Mn, and PPP/MTX groups. Consistently, quantitative analysis demonstrated that treatment with PPP/MTX–Mn significantly reduced tumor weight (Fig. 6B) and tumor volume (Fig. 6C) compared with the other groups, indicating enhanced antitumor activity of the MTX–Mn-loaded hydrogel.

To further assess tumor cell proliferation *in vivo*, Ki-67 immunohistochemical staining was performed. As shown in Fig. 6D, the PPP/MTX–Mn group exhibited a marked decrease in Ki-67 positive cells compared with the control and single-agent groups, suggesting effective suppression of tumor cell proliferation. Quantification of Ki-67 positivity further confirmed a significantly reduced proliferative index in the PPP/MTX–Mn group (Fig. 6D). The biosafety of the hydrogel formulations was evaluated by histological analysis of major organs. Hematoxylin and eosin (HE) staining of the heart, liver, spleen, lungs, and kidneys showed no obvious pathological abnormalities in any treatment group (Fig. 6E), indicating good *in vivo* biocompatibility and minimal systemic toxicity of the PPP/MTX–Mn hydrogel.

However, this study has several limitations that should be acknowledged, and there are clear avenues for improvement in future research. First, only male C57BL/6 mice were used in the *in vivo* experiments, which limits the generalizability of the results, as sex differences—particularly hormonal influences—may affect therapeutic efficacy and safety; therefore, future studies should include both male and female models. Furthermore, the current study lacks comprehensive toxicological evaluations, such as mutagenicity and immunotoxicity assessments, which are necessary to more fully characterize the safety profile of the developed system. Additionally, although short-term stability of MTX-Zn over 7 days was investigated, longer-term stability studies are still needed to better evaluate the sustained performance, potential degradation, and overall safety of the drug delivery system over extended periods.

## 4 Conclusions

In summary, we developed a thermosensitive PLGA–PEG–PLGA hydrogel incorporating methotrexate–manganese (MTX–Mn) coordination nanoparticles for localized melanoma therapy. MTX–Mn exhibited enhanced antitumor activity by inhibiting melanoma cell proliferation and migration, inducing cell-cycle arrest and apoptosis, and triggering severe mitochondrial dysfunction through excessive ROS generation and mitochondrial membrane potential collapse. The injectable hydrogel enabled *in situ* gelation, sustained drug release, and prolonged local retention of MTX–Mn. *In vivo* studies demonstrated that peritumoral administration of the MTX–Mn-loaded hydrogel effectively suppressed tumor growth with good biosafety. This work highlights a promising strategy that combines metal-drug coordination nanomedicine with thermosensitive hydrogels for safe and effective localized melanoma treatment.

## Ethical statement

All animal experiments were performed in compliance with the relevant laws and guidelines and followed the institutional guidelines of Central South University. All procedures were conducted in accordance with the Guidelines for the Care and Use of Laboratory Animals of Central South University. The experimental protocols were reviewed and approved by the Animal Ethics Committee of Central South University.

## Author contributions

Xiaolong Xu: investigation, methodology, validation, data curation, formal analysis, writing-original draft. Yuze Zhou, Junhao Chen, Lujing Fei: methodology, data curation, formal analysis. Yuze Zhou: validation. Lujing Fei: review, validation. Min Qi: conceptualization, supervision, review and editing, funding acquisition, project administration.

## Conflicts of interest

The authors declare that they have no known competing financial interests or personal relationships that could have appeared to influence the work reported in this paper.

## Supplementary Material

RA-016-D6RA01096B-s001

## Data Availability

The raw data required to reproduce these findings are available from the corresponding author upon reasonable request. Supplementary information (SI) is available. See DOI: https://doi.org/10.1039/d6ra01096b.
