# Supplementary material for: An injectable thermosensitive PLGA–PEG–PLGA hydrogel integrated with coordination-driven self-assembled MTX–Mn nanoparticles for enhanced melanoma therapy via mitochondrial dysfunction
Source: RSC Adv. 2026 Apr 22;16(23):20895–907. doi: 10.1039/d6ra01096b (PMC13101956; doi:10.1039/d6ra01096b)
Supplement: RA-016-D6RA01096B-s001 [file RA-016-D6RA01096B-s001.pdf]

Supplementary Information (SI) for RSC Advances.
This journal is © The Royal Society of Chemistry 2026

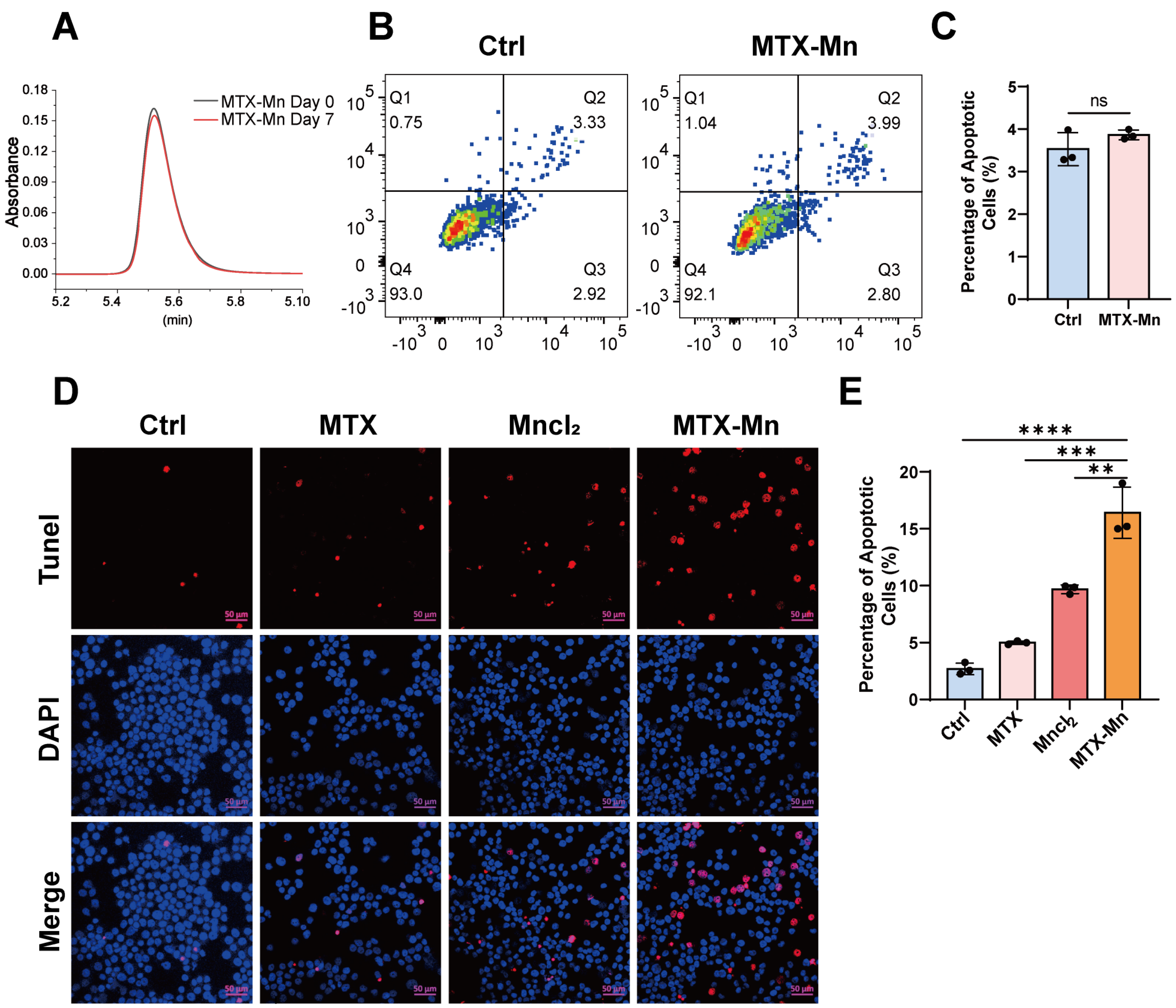

Fig. S1 (A) HPLC analysis showing the chemical stability of MTX-Mn over seven days, with no significant degradation detected. (B) Representative Annexin V-FITC/PI flow cytometry plots of NIH/3T3 fibroblasts treated with MTX-Mn. (C) Quantitative analysis of apoptotic NIH/3T3 cells. MTX-Mn treatment showed a slight increase in apoptosis compared with the control group; however, no statistically significant difference was observed. (D) Representative fluorescence images of TUNEL staining in B16F10 melanoma cells. (E) Quantitative analysis of apoptotic B16F10 cells based on TUNEL staining. Data are presented as mean ± SD (n = 3). Statistical significance is indicated as $**P < 0.01$, $***P < 0.001$, and $****P < 0.0001$; ns, not significant.
